# Detection of Human Cytomegalovirus Proteins in Paraffin-Embedded Breast Cancer Tissue Specimens—A Novel, Automated Immunohistochemical Staining Protocol

**DOI:** 10.3390/microorganisms9051059

**Published:** 2021-05-13

**Authors:** Joel Touma, Yan Liu, Afsar Rahbar, Mattia Russel Pantalone, Nerea Martin Almazan, Katja Vetvik, Cecilia Söderberg-Nauclér, Jürgen Geisler, Torill Sauer

**Affiliations:** 1 Department of Breast and Endocrine Surgery, Akershus University Hospital (AHUS), 1478 Nordbyhagen, Norway; joel.touma@ahus.no (J.T.); katja@vetvik.org (K.V.); 2 Institute of Clinical Medicine, University of Oslo, Campus Akershus University Hospital (AHUS), 1478 Nordbyhagen, Norway; torill.sauer@medisin.uio.no; 3 Department of Clinical Molecular Biology, University of Oslo, 0315 Oslo, Norway; yan.liu@medisin.uio.no; 4 Department of Clinical Molecular Biology (EpiGen), Akershus University Hospital (AHUS), 1478 Nordbyhagen, Norway; 5 Microbial Pathogenesis Unit, Department of Medicine, BioClinicum, Karolinska Institute, 17177 Solna, Sweden; afsar.rahbar@ki.se (A.R.); mattia.pantalone@ki.se (M.R.P.); nerea.martin.almazan@ki.se (N.M.A.); 6 Department of Neurology, Karolinska University Hospital, 17177 Solna, Sweden; 7 Department of Oncology, Akershus University Hospital (AHUS), 1478 Nordbyhagen, Norway; 8 Department of Pathology, Akershus University Hospital (AHUS), 1478 Nordbyhagen, Norway

**Keywords:** human cytomegalovirus, HCMV, breast cancer, immunohistochemical staining, IHC

## Abstract

Emerging evidence supports a significant association between human cytomegalovirus (HCMV) and human malignancies, suggesting HCMV as a human oncomodulatory virus. HCMV gene products are found in >90% of breast cancer tumors and seem to be correlated with more aggressive disease. The definitive diagnosis of HCMV relies on identification of virus inclusions and/or viral proteins by different techniques including immunohistochemical staining. In order to reduce biases and improve clinical value of HCMV diagnostics in oncological pathology, automation of the procedure is needed and this was the purpose of this study. Tumor specimens from 115 patients treated for primary breast cancer at Akershus University Hospital in Norway were available for the validation of the staining method in this retrospective study. We demonstrate that our method is highly sensitive and delivers excellent reproducibility for staining of HCMV late antigen (LA), which makes this method useful for future routine diagnostics and scientific applications.

## 1. Introduction

Breast cancer is the most commonly diagnosed malignancy and leading cause of cancer related death among women worldwide, where 1 out 8 women are at risk of developing the disease during their lifetime [1], this malignancy is a major public health problem and global data predict incidence and mortality to still be on the rise [2]. Early diagnosis of breast cancer is a prerequisite for successful treatment and a high survival rate [3]. Some of the known risk factors for this malignancy include age, early age at menarche, late age of menopause, hormone replacement therapy usage, and positive family history [4]. However, up to 80% of patients diagnosed with breast cancer do not present with any of the known risk factors and the disease is considered sporadic [4]. In other words, the etiology remains unclear for the majority of breast cancer patients and it is therefore important to further explore additional risk factors. Recently, infectious pathogens have emerged as potential contributors to breast cancer carcinogenesis and onco-modulation [5,6,7].

### 1.1. Human Onco-Viruses

The first evidence suggesting that some human cancers could be caused by viral infections begun to emerge in 1964 [8]. Since then, a number of different mechanisms has been linked to virus induced tumor development. Currently there are seven human viruses that are classified as oncogenic: Human papillomavirus (HPV), Hepatitis B virus (HBV), Hepatitis C virus (HCV), Epstein–Barr virus (EBV), Kaposi sarcoma-associated herpesvirus (KSHV), Merkel cell polyomavirus (MCPyV), and Human T-lymphotrophic virus 1 (HTLV-1) [8]. Some of the human and animal oncogenic viruses, such as mouse mammary tumor virus (MMTV), bovine leukemia virus (BLV), HPVs, and EBV are implied to specifically play a role in breast cancer development [9].

Considering the dramatic success of established vaccines and antivirals in reducing the incidence of tumors of viral origin, virus-induced cancers present actionable opportunities for prophylaxis, diagnostics, and therapy [10]. In this context, the interest for the widespread and common human cytomegalovirus (HCMV) has risen during the past decades, as reports linking HCMV with a variety of malignancies, including breast cancer, continue to emerge [5,6,7,11].

### 1.2. Human Cytomegalovirus (HCMV)

HCMV, also known as human herpes virus 5 (HHV-5), is a member of the β-herpesvirus family. This ubiquitous, opportunistic DNA virus, infects up to 90% of the world’s population and establishes a life-long persistence within the host [12]. Although considered harmless in healthy immunocompetent individuals, reactivation of the latent HCMV is a dreaded complication in immunocompromised patients, causing significant morbidity and mortality [13]. Reactivation of latent HCMV can be triggered by inflammatory processes and the virus becomes disseminated to peripheral organs by monocytes/macrophages or dendritic cells, which are inflammatory cells serving as carriers for the virus to sites of infection or inflammation [14,15] where these cells can transmit the virus to different cell types. Reactivated HCMV can infect and replicate in a broad number of cell types, potentially causing disease in almost all organs in the human body [14,16].

Between individuals, HCMV has a large variation in transmission patterns including placental transfer, breast-feeding, saliva, sexual contact, blood transfusion, and solid-organ or stem-cell transplantation. Breast milk, close personal contact, and sexual contact are considered a primary route of transmissions in humans worldwide. After primary infection, HCMV establishes latency in the hematopoietic progenitor cell population in the bone marrow [14]. The breast epithelium also serves as a site for persistent infection and/or viral reactivation [11]. Hamprecht et al. showed that 90% of breast milk samples from HCMV seropositive women contain infectious virus allowing for infection of infants early in life, and results in 30% HCMV prevalence in children at one year of age [17]. Upon entry into the host cell, HCMV can exhibit immunosuppressive effects through disabling cellular intrinsic and innate immune responses and avoiding recognition and elimination by the adaptive immune system, which leads to enhanced virus and cell survival [18]. When the viral DNA has entered the cell nucleus, the first genes that are expressed are the genes coding for immediate early (IE) proteins [19]. The HCMV genes are expressed in three distinct phases after infection to ensure viral replication, which result in the production of immediate early (IE), early (E), and late (LA) proteins [20]. IE proteins function as viral transcription factors, while E and LA proteins are structural proteins forming the virus particle [20]. HCMV tightly modulates its own gene expression during a cascade of events that are controlled through complex regulatory mechanisms, first resulting in activation of the major immediate-early promoter (MIEP) and production of IE proteins. The IE proteins further regulate viral gene expression as well as host gene expression in order to establish lytic infection or viral re-activation from latency [21].

### 1.3. HCMV in Cancer

HCMV has a significant association to human malignancies. The virus has been found in >90% of glioblastoma, medulloblastoma, neuroblastoma, colon cancer, prostate cancer, ovarian cancer, Hodgkin and Non-Hodgkin Lymphoma, and breast cancer [22,23,24,25,26,27,28,29]. HCMV proteins are found in >90% of breast cancer tumors and in >90% of metastatic deposits from breast and colon cancer [30]. Higher viral activity as depicted by enhanced viral protein presence in the tissue, correlates with the most aggressive breast cancer phenotype, triple-negative breast cancer [31]. As these tumors are notoriously resistant to therapy, the significant presence of an active HCMV infection in breast cancer, needs further investigation as it may provide new targets of therapy [5].

HCMV IE gene products have been shown to directly and indirectly interfere with key cellular pathways of cancer biology such as causing chromosomal aberrations, DNA damage and disruption of DNA repair mechanisms, resulting in genetic instability and mutations in infected cells, all of which are hallmarks of cancer cells [32,33,34]. HCMV proteins can also induce inflammation and angiogenesis, dysregulate cell cycle progression, block apoptotic pathways, inhibit tumor suppressor functions, and alter cellular metabolism to the Warburg effect [35,36,37,38,39]. Given the accessibility that HCMV has to a variety of cell types and tissues, its ability to manipulate the immune response and fueling the development of genetic mutations within the cell, this virus exhibits key features required for oncogenic properties of a tumor associated virus. Emerging evidence from recent publications provide support that HCMV fulfills the requirements of all hallmarks of cancer, and suggest that it should be considered as a human oncogenic virus [40,41,42,43].

Considering the wide presence of HCMV in different tumor types, this virus may represent a potential target of therapy for HCMV positive malignancies, including breast cancer. Retrospective clinical trials have shown that antiviral therapy with valganciclovir as an add on to standard therapy seems to improve survival in patients with glioblastoma [44,45], and also dendritic cells vaccination shows promising results in these patients [46].

### 1.4. HCMV Diagnostics

HCMV infection is defined by the presence of virus without symptoms, while HCMV disease is an HCMV infection accompanied by clinical signs and symptoms from organ engagement of the infection [47]. HCMV disease can present as pneumonitis, gastrointestinal disease, hepatitis, retinitis, encephalitis, nephritis, cystitis, myocarditis, and pancreatitis [48]. Serological methods and PCR-based techniques are widely used clinically to investigate primary infection, previous exposure, reactivation and response to therapy [49]. However, the definitive diagnosis of tissue-invasive disease relies on identification of HCMV inclusions or HCMV antigens in tissue specimens by immunohistochemistry (IHC) or other staining protocols [50,51]. Table 1 summarizes different techniques currently in use to identify and confirm HCMV-infection in different specimens [49,50,52,53].

In general, IHC techniques include the following steps: (1) deparaffinization of tissue sections in order for aqueous solutions to penetrate into the tissue, (2) antigen retrieval by demasking the antigenic epitopes, (3) extinguishing endogenous enzymes that could react with IHC reagents giving false positive results, (4) blocking of non-specific binding sites, (5) binding with primary antibodies specifically targeting the epitopes of interest, (6) binding of conjugated secondary antibodies to the primary antibodies, (7) development of positive signal with chromogen substrate, (8) counterstaining, (9) dehydrating, and (10) mounting [54].

Cobbs et al. were first to present evidence of the presence of HCMV proteins in different types of tumor tissue, including breast cancer, using IHC [25,55,56,57]. They used extensively optimized and highly sensitive manual IHC protocols that deviated significantly from the routine HCMV immunodetection used in for instance for detection of HCMV in tissue specimens obtained from HCMV infected AIDS or transplant patients [55,56,57,58]. Taher and Rahbar et al. further optimized a more sensitive manual protocol for detection of HCMV proteins in breast cancer tissue specimens [27,31,59].

Detection of HCMV proteins in tumor tissues is not just a random rare finding. Optimized staining protocols detect HCMV proteins in a majority of tissue specimens from a variety of tumors. A higher load of HCMV proteins has been associated with increased inflammation in colon and breast cancer, and with reduced expression of estrogen and progesterone receptors in breast cancer, which are known as poor prognostic factors [31,56,59]. High CMV protein levels are associated with shorter overall survival in patients with glioblastoma [60,61]. These observations suggest that HCMV may affect the malignancy grade of tumors and shorten life expectancy of patients.

Immunostaining is generally performed according to a multi-step laboratory manual, which is often a considerably time-consuming and complex process that benefits from an automated procedure. Robots have been evolved to carry out the different steps of reagent application and washing, and to further manage slide labeling, baking, deparaffinization, rehydration, antigen retrieval, application and incubation of antibodies, development of signals, cover-slipping, and digital image analysis. Due to the convincing advantages that automation offered, adoption of automated IHC staining platforms are today widespread in clinical laboratories with Good Laboratory Practice (GLP) [62].

## 2. Materials and Methods

For HCMV, one of the main challenges for adapting a manual IHC protocol for HCMV-detection into an automated system are the use of highly specific reagents from one manufacturer, and instruments from other non-related companies. The purpose of this study was to adapt a strictly defined manual HCMV staining protocol for breast tissue sections, into an open system IHC robot to improve the results in terms of quality, sensitivity, specificity, and reproducibility.

### 2.1. Patient Samples

Formalin-fixed paraffin-embedded (FFPE), breast tumor samples and metastases obtained from 115 patients treated for breast cancer between 1996 and 2010 at Akershus University Hospital, Norway were collected for this retrospective study. All available samples were subjected for validation by the automated IHC staining method for detection of HCMV proteins. Ethical permission was approved by the regional ethical committee of south-east Norway (No: 2014-895).

For each patient, three tissue sections were used for detection of HCMV IE, HCMV LA and Cytokeratin 20 (CK20), the latter serving as negative control as the irrelevant protein with the same type of isotype as antibodies used to detect HCMV proteins. One breast carcinoma with known positivity from previous studies, was used as positive control for both anti-HCMV immediate early and late antibodies, respectively, in each run of the procedure.

### 2.2. Reagents

The following antibodies were used: Anti-Cytomegalovirus, immediate early proteins (1:200 MAB810R, Millipore, clone 8B1.2, IgG2a) targeting the IE72 (IE1) and IE86 (IE2) proteins; Anti-Cytomegalovirus, late protein (1:200 MAB8127, Millipore, clone 1G5.2, IgG2a); Anti-Cytokeratin 20 (ready to use, IR777, Dako, Clone Ks20.8, IgG2a) was used as negative control.

### 2.3. Immunohistochemical Staining

For optimizing IHC automation, we used a lung carcinoma sample with known positivity for HCMV as a control. The antibodies to HCMV IE and HCMV LA, were diluted in a serial of 1:100, 1:200; 1:400; 1:800 dilutions according to the manufacturer’s instructions, both high pH and low pH antigen retrieval solutions were tested in combination with all serial dilutions. Selection of reagents and incubation times were adjusted to achieve strong signal while eliminating unspecific binding. Further testing was then performed on two breast carcinomas with known positivity for HCMV, for validation. Finally, automation IHC protocols for HCMV were set up according to the best signal to background ratio on breast carcinoma as follows.

The breast cancer specimens were cut in 4 µm thick sections, hematoxylin and eosin stain was done with routine pathology procedure for histopathological evaluation. Immunohistochemistry was performed with labelled polymer and enhanced polymer systems Dako Envision™ system (Dako, Agilent Technologies, Santa Clara, CA, USA) on Dako autostainer link 48 platform.

In detail, antigen retrieval was achieved in a PT-Link station by immersion into either EnVision™ FLEX Target Retrieval Solution, high pH (K8004, Dako) for both HCMV-LA and CK20 or EnVision™ FLEX Target Retrieval Solution, low pH (K8005, Dako) for IE and run on a heating program at 97 °C for 20 min in PT-Link. Endogenous peroxidase activity was quenched by incubating the slides in EnVision™ FLEX peroxidase blocking reagent (K8000, Dako) for 5 min; non-specific staining was inhibited by background sniper (BC-BS966L, Biocare) for 15 min; primary antibodies were diluted in EnVision™ FLEX Antibody Diluent (K8006, Dako); and slides were incubated with both anti-HCMV immediate early (dilution 1:200) and late antibodies (dilution 1:200), respectively for 60 min and anti-CK20 antibody (Ready to use) for 20 min at room temperature. Following incubation with the ready-to-use secondary buffered solution (k8002, EnVision FLEX /HRP, Dako) for 20 min at room temperature, sections were treated with 3,30-diamino-benzidine tetrahydrochloride (DAB) solution (1drop of EnVision™ FLEX DAB+ Chromogen diluted into 1 mL EnVision™ FLEX Substrate Buffer, Dako) for 10 min at room temperature and counterstained with Hematoxylin (Link) (k8008, Dako) for 5 min. After dehydration, coverslips were mounted on top of sections by automated cover slipper (ClearVue™, Thermo Scientific, Waltham, MA, USA). Whole slide images were acquired by digital pathology slide scanner at 20X (ScanScope AT, Aperio, Leica, Buffalo Grove, IL, USA).

### 2.4. DNA Extraction and Quantitative PCR (qPCR)

Paraffin embedded (FFPE) breast cancer sections were deparaffinized in xylene 2 times for 5 min each, followed by rinsing in 100% ETOH for 3 min. Sections were then scraped off slides and DNA was extracted with QIAamp DNA FFPE Tissue Kit (56404, Qiagen) according to the manufacturer’s instructions.

Extracted DNA was quantified using Nanodrop 2000 (Thermo Fisher Scientific). Approximately 100 ng of DNA per sample was amplified with TaqMan Fast Universal PCR Master Mix (Life Technologies) using the following specific TaqMan probes: HCMV IE DNA, HCMV pp65 (custom made TaqMan assays, Applied Biosystems) the primers were designed according to Kabelitz et al. [63] and human β2-microglobulin (B2M, assay ID, Hs00984230_m1) (Life Technologies, Waltham, MA, USA) was used as housekeeping gene. The PCR was performed using a 7900HT Fast Real-Time PCR system (Applied Biosystems, Waltham, MA, USA). Positive and negative controls were DNA from HCMV strain VR1814 infected and uninfected human fibroblasts cell line (MRC5, ATCC).

### 2.5. Viral Infection of Breast Cancer Cells in Culture

Breast cancer cell lines MCF-7 (ER/PR positive but HER2 negative), MDA-MB-231 (ER/PR/HER-2 negative), SK-BR-3 (overexpression of the HER2/c-erb-2 gene product) from ATCC, were cultured in RPMI 1640 medium supplemented with 10% fetal bovine serum (FBS), 100 U/mL of penicillin and 100 µg/mL of streptomycin and maintained in a 37 °C incubator with 5% CO_2_. Viral stocks of HCMV VR1814 strain were prepared through virus propagation in human umbilical vein endothelial cells (HUVEC) at low passage and ultracentrifugation of supernatants. Breast cancer cell lines were infected with HCMV strain VR1814 at a multiplicity of infection (MOI) of 3, and collected for analyses at 1, 5-, and 10-days post infection (dpi).

### 2.6. RNA Extraction and Quantitative Reverse Transcription PCR (RT-qPCR)

MCF-7 and MDA-MB-231 and SK-BR-3 cells were infected with HCMV VR1814 at multiplicity of infection (MOI) of 3, medium was changed after 2 h and cells were washed with PBS and cultured for 1-, 5-, 10-, and 15-days post-infection (dpi), when cells were collected for further analyses. RNA was extracted from lysed cells using TRIzol™ Reagent (Thermo Fisher Scientific, Waltham, MA, USA) method according to manufacturer’s instructions and cDNA was synthesized using random primers and the high-capacity cDNA reverse transcription kit (Applied Biosystems, Waltham, MA, USA). Gene expression levels were quantified by real-time PCR using TaqMan Fast Universal PCR Master Mix (Life Technologies) and the following specific TaqMan probes: HCMV-IE cDNA, HCMV pp65 (custom made TaqMan assays, Applied Biosystems, the primers were designed according to Kabelitz et al. [63] and human β2-microglobulin (B2M, assay ID, Hs00984230_m1) (Life Technologies) was used as housekeeping gene. The PCR was performed using a 7900HT Fast Real-Time PCR system (Applied Biosystems, Waltham, MA, USA). The endogenous control B2M was used for normalization and relative expression was determined by the ΔCt method. Experiments were performed in triplicates.

## 3. Results

**Figure 1:**
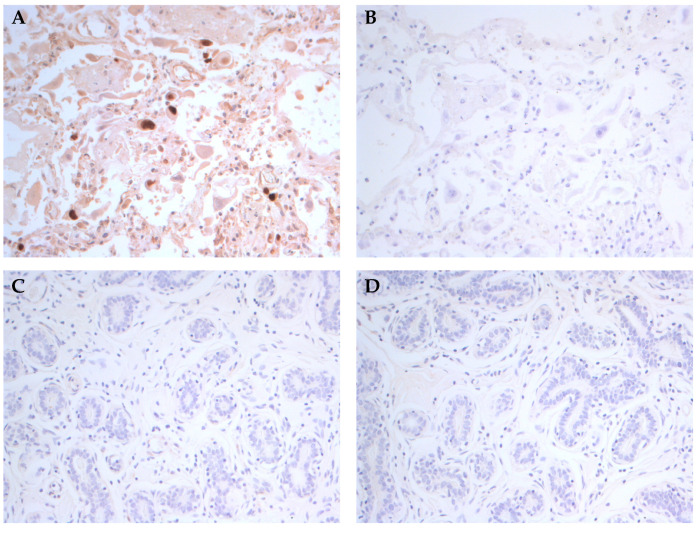
Expression of HCMV-IE protein in HCMV infected lung tissue and normal non-infected breast tissue sections by automated IHC. HCMV-IE protein (brown color) was detected in HCMV infected lung tissue section (**A**) but not in uninfected normal breast tissues (**C**) by the use of an automated IHC approach. Primary antibody was omitted in both tissues sections and served as internal negative controls for IHC method (**B**,**D**). Original magnification ×20.

We developed an automated protocol for staining of HCMV IE and LA proteins in breast cancer tissue specimens and obtained high quality images, detecting HCMV LA in 75% of our specimens. HCMV positive lung tissue and normal non-HCMV infected breast tissue were used a positive and negative controls, respectively (Figure 1).

**Figure 2:**
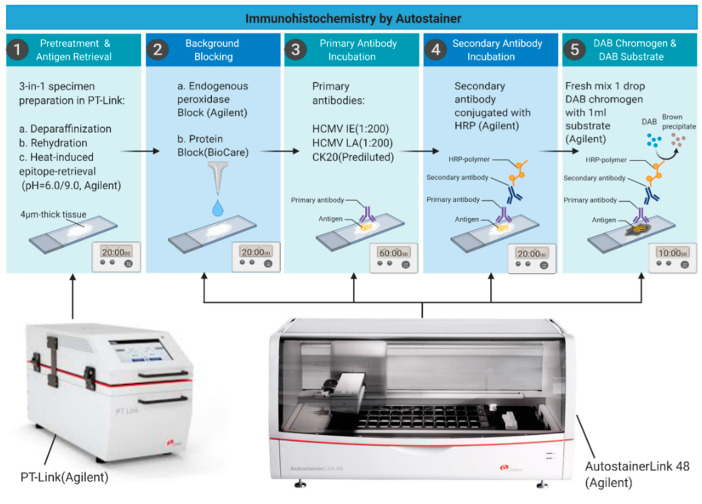
Schematic presentation of the automated IHC process. Created with BioRender.com (accessed on 5 April 2021).

Our novel fully automated HCMV IHC procedure (summarized in Figure 2) was in our hands, a fast and reliable method providing reproducible results. The whole IHC procedure, from the antigen retrieval step until mounting of the slides, in a total of 345 slides, was done in four working days only, in contrast to the former manual protocol that would have consumed approximately 6–8 weeks for staining of similar number of slides.

The steps for pretreatment and antigen retrieval, background blocking, primary antibody incubation, secondary antibody incubation, development of signal and mounting of slides, were all included in this automated protocol and performed by a robot. Out of the 115 breast cancer patient specimens collected, 103 were primary tumor tissue and 8 lymph node metastases. Specimens not containing tumor tissue (*n* = 1), but consisting of carcinoma in situ (*n* = 2) and normal tissue (*n* = 1), were excluded from the evaluation (4 patient samples in total).

**Figure 3:**
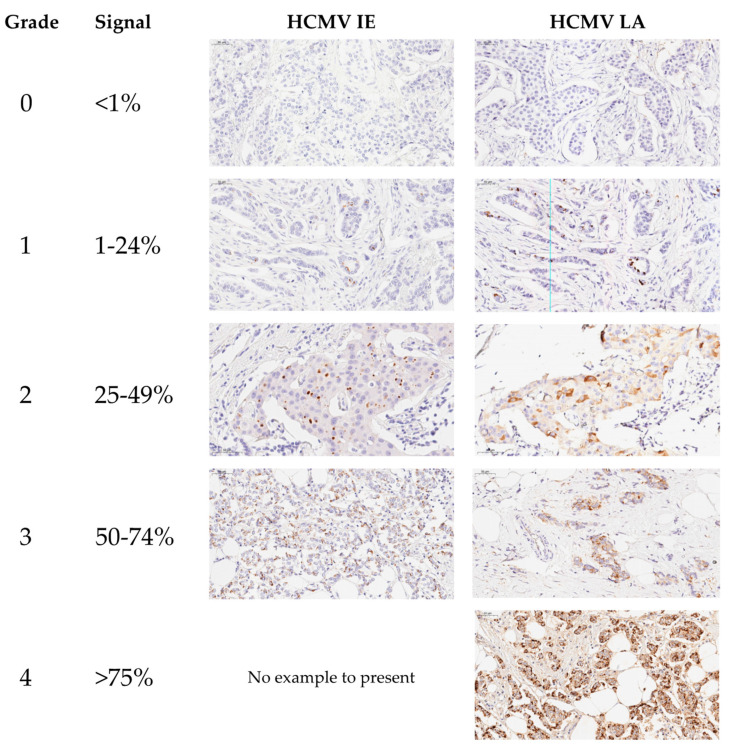
Demonstration of HCMV IE and LA in breast cancer tissue specimens by using an automated IHC approach. Approximately estimated number of the tumor cells expressing HCMV-IE and LA within the breast tumor sections are scored as 0; <1%, 1; 1–24%, 2; 25–49%, 3; 50–74%, and 4; >75%. HCMV IE and LA (brown color) are expressed in the cytoplasm of the tumor cells. There was no HCMV IE grade 4 among our specimens. Original magnification ×20.

HCMV protein expression in the primary tumor tissue and lymph node metastasis specimens was evaluated by an experienced breast cancer pathologist at Akershus University Hospital and scored according to a system of grade 0–4, where zero expresses <1% signal of HCMV expression in the tissue specimen examined, one expresses 1–24%, two expresses 25–49%, three expresses 50–74%, and four expresses >75% positivity of HCMV IE or LA within the tissue specimen, respectively (Figure 3).

We found that 83 out of 111 (~75%) of the breast cancer patient samples were positive for HCMV LA in tumor tissue and/or lymph node metastasis from breast cancer, and 9 out of 111 (~8%) samples were positive for HCMV IE proteins (Table 2). HCMV IE and LA expression was consistently localized in the cytoplasm of the cell as well as a typical endothelial lining in some specimens, no nuclear staining was observed throughout the evaluation process.

In 17 out of the 83 patients with LA positivity, LA expression was found both within the tumor and the carcinoma in situ compartment of the tumor. HCMV LA proteins were also found in non-tumor tissue surrounding the tumor in 20 patients, and in 6 patients LA expression was found in tumor, carcinoma in situ and in non-tumor adjacent tissue. Furthermore, HCMV was found solely in non-tumor tissue in six patient samples, with no expression in the tumor cells nor carcinoma in situ compartment, five patient samples expressed IE positivity and one sample expressed LA proteins. Throughout the evaluation process, we obtained high-quality images with no background staining and a highly specific visualization of the distribution of HCMV protein positive cells in breast cancer tissue specimens.

To confirm the results obtained by IHC, we extracted DNA from 20 paraffin embedded breast cancer tissues and performed a qPCR for HCMV IE and pp65. Of the 20 tissue samples, 6 were positive for HCMV-IE DNA, and 1was positive for pp65 but negative for IE DNA. All seven tissue specimens positive by qPCR were also strongly positive for HCMV-LA proteins by IHC. One of the HCMV-IE DNA positive samples was also HCMV-IE and LA positive by IHC.

**Figure 4:**
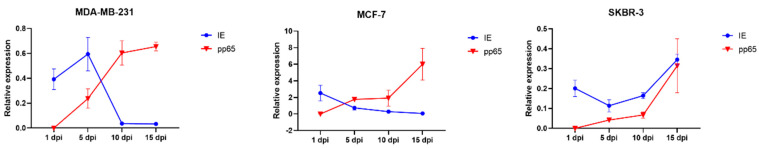
Detection of HCMV transcripts in three different breast cancer cell lines over time of infection. While expression of HCMV-IE transcripts disappeared over time in MDA-MB-231 and MCF-7 cell lines, expression of HCMV-pp65 transcripts persisted and were enhanced over time of infection. Expression of HCMV-IE and HCMV-pp65 transcripts were also detectable in the SK-BR-3 cell line over time of infection and increased at 15 dpi. Transcript levels of HCMV-IE and HCMV-pp65 were normalized to housekeeping gene B2M at different time points (1, 5, 10, and 15 dpi) in three different breast cancer cell lines (MDA-MB 231 on the left, MCF-7 center, and SK-BR-3 on the right). Assays were performed in three separate experiments.

In order to clarify the lack of HCMV IE and the presence of HCMV LA in breast tumor tissue sections, we analyzed the presence of HCMV IE and LA in three different in vitro *HCMV*-infected breast cancer cell lines over time of infection. PCR analysis showed that HCMV IE transcripts were expressed in all three cell lines at 1 and 5 dpi; however, at 10 and 15 dpi IE transcript levels were low in MDA-MB-231 and MCF-7. Interestingly, levels of IE transcripts remained detectable also at later time points in SK-BR-3 cells. Late antigen pp65 is expressed in all three cell lines at 5 dpi and persisted and even increasing at later time points reaching the highest levels at 15 dpi (Figure 4).

## 4. Discussion

Through continuous optimization, a reliable automated IHC protocol was established for the pivotal HCMV IE and LA proteins. Using this method, we found distinct cytoplasmic staining of mainly LA but also IE proteins without background staining in the majority of our breast cancer samples. We found that 83 out of 111 (~75%) of the breast cancer patient samples were positive for HCMV LA and 9 out of 111 (~8%) samples were positive for HCMV IE proteins (Table 2). Similar observations were made in HCMV infected tumor cells in vitro. While IE was expressed at early times of infection and decreasing already after 5 dpi, pp65 was still expressed and even enhanced at 15 days post infection. This suggests that long term HCMV infected tumor cells may preferentially express late genes, just as we observed in clinical tumor samples. In tissue samples, we were able to detect IE DNA in 6 out of 20 breast cancer tissue samples analyzed by qPCR, but only one tissue sample was positive for pp65. This is an expected finding as PCR analysis of tumor tissue specimens show lower sensitivity of detection of HCMV DNA than immunohistochemistry analyses [64]. We further speculate that different epigenetic control mechanisms in the HCMV MIE promoter may affect the transcription of HCMV genes in the early and late stages of infection in these three breast cancer cell lines. Further studies are needed to elucidate the complex regulatory mechanisms behind attenuation of HCMV IE gene expression and sustained late gene expression in these cell lines.

According to our results obtained by automated IHC, the number of tissue sections positive for HCMV IE proteins was lower than anticipated when comparing with the literature and including our own experience using manual staining [22,23,24,25,26,27,28,31]. This discrepancy may partly be explained by the individual technical steps such as primary antibody dilution, variation in incubation time for primary antibodies, difference in pH, temperature and treatment of the tissue with antigen retrieval buffer in manual versus automated methods. Moreover, another possible reason might be that considerably low levels of HCMV IE protein expression can simply not be detected due to level below detection limit, while HCMV LA was more easily detected, at the thresholds set to limit non-specific staining. The expression of IE proteins is essential for the expression of viral late proteins [65]. Therefore, IE proteins have likely been expressed before the late proteins appeared and then remained in a chronic phase of infection, as was observed in tumor cells infected with HCMV in vitro.

Expression of HCMV IE and LA proteins was consistently localized in the cytoplasm of the cell as well as a typical endothelial lining in some specimens, no nuclear staining was observed throughout the evaluation process. Could automation of evaluation of samples further improve this diagnostic method?

Automated quantification of IHC staining has been investigated in a number of studies, and in breast cancer, it has been done for all routine markers [66,67,68,69,70,71]. Although detection and quantification of HCMV in cancer specimens have the potential to offer clinically valuable information, the establishment of standardized and reliable diagnostic methods is a matter in need of consensus. Our aim was to establish an automated IHC protocol for HCMV diagnostics in breast tumor tissue sections.

There is no doubt that automated quantification has less variability compared to the manual scoring. Nevertheless, all guidelines for therapeutic markers, in breast cancer and other cancers accept manual scoring. The vast majority of pathology departments worldwide, including Europe, have manual scoring as standard procedure. Automated scoring requires a perfect slide in all aspects, both technically and immunohistochemical staining quality. This requires demanding lab work and is time consuming. For this study, however, the main focus was the IHC procedure, a more exact quantification will be dealt with later.

An infection at early stage of pre-malignant transformation may contribute to the carcinogenic process considering that HCMV can induce all the hallmarks of cancer. Recently, a clinically isolated HCMV strain (HCMV-DB) was shown to induce oncogenic properties in infected human mammary epithelial cells [33]. Such HCMV-transformed cells turned to an aggressive triple negative phenotype in vitro and were able to form tumors when injected in NSG mice [33,72]. In another murine model, latent cytomegalovirus infection was associated with enhanced metastases [73]. A recently published study also indicates that some clinical HCMV strains carry the potential to transform human mammary epithelial cells and fit with a blastomere-like model of oncogenesis. This may be of relevance in the pathophysiology of breast cancer, especially in those with poor prognosis [74]. Taken together, experimental evidence suggests a role of HCMV in different stages of cancer development and highlight the possibility that not only an active infection with strong IE expression but also a late stage of infection or even a latent infection may affect disease progression.

Some previous publications investigating the presence of HCMV proteins in tumor specimens have showed inconsistent results, possibly due to difficulties in detecting viral gene products when not using optimized protocols [75,76,77,78]. In a consensus report published by Dziurzynski et al., it was discussed that an optimized IHC protocol is crucial to use for detection of HCMV proteins in tumor tissues [64]. Critical steps in an IHC protocol include, embedding and sectioning of specimens, choice of primary antibodies fit for use in IHC, optimized retrieval solutions with correct pH, incubation time, and temperature. It is essential to titrate the antibody dilution for optimal detection of HCMV IE and LA proteins in tumor cells. In our own experience, longer incubation time with the IE specific antibody yield higher grade of cytoplasmic staining while corresponding isotype control antibody does not. The relevance of this observation is today uncertain. HCMV IE proteins are localized to the nucleus in in vitro infected cells in culture and in tissue samples from patients with acute HCMV diseases. Cytoplasmic staining patterns are only observed under certain conditions in in vitro infected cells, but represent a common feature of HCMV positive tumor cells. Which isoforms of IE proteins these correspond to is at present unclear and also if they represent the positive signals that are found in tissue specimens after longer incubation times. This emphasizes the importance of diagnostic methods to be optimized, standardized, and quality assured, hence a main purpose of this study in which we used strict optimization to exclude potential background staining.

A key point in immunohistochemical staining is demasking the tissue from the formalin fixation and the need to disrupt the tertiary structure of the antigens to get access to the specific epitope. In the manual method this was obtained by using a pressure cooker, which later on was replaced by microwave heating for a more even temperature, also allowing this step to be possible to employ in an automated protocol. Furthermore, this is a specific heating step in the automated procedure that is fixed and optimized by the operator to fulfill the specific requirements of the antibody. When all steps are performed by a robot, it is not only time-preserving but also quality consistent, avoiding possible irregularities and unwanted errors that could appear during manual procedures.

Using our improved and automated method, we have managed to overcome the previous challenges with automation, and identify HCMV proteins in breast cancer tissue specimens, with high sensitivity and specificity to the morphological distribution of the virus. Our results presented here significantly concur with previous publications confirming a high prevalence of HCMV in primary breast cancer tumors and metastatic descents from breast cancer [6,7,11,27,31,59].

Overall, our automated method provides optimal quality images with high sensitivity to HCMV IE and LA expression in a highly standardized manner, even allowing for semi-quantification analysis.

## 5. Conclusions

Due to the increased interest in HCMV as an onco-modulatory virus and its potential role in breast cancer carcinogenesis, an automated IHC method to be used by diagnostic laboratories is warranted. We developed a fast and reliable method that gives reproducible staining results for mainly HCMV LA proteins, which allows for quantification of these viral antigens. Thus, this method will be useful for both clinical routine staining and for scientific purposes to further evaluate the role of HCMV in breast cancer.

## Figures and Tables

**Table 1:** Summary of conventional techniques used for detection of HCMV infection in different specimens.

Method	Specimens	Comments
Serology	Blood	The presence of IgG determines that a patient has had HCMV infection in the past and is considered a carrier of latent virus. Detection of IgM without detectable IgG indicates acute primary infection, while detection of both IgM and IgG indicates reactivated HCMV infection.
Antigenemia	Blood	Use of monoclonal antibodies to detect the presence of HCMV pp65 in neutrophils during the early period of the virus replication cycle. Reported as number of pp65-positive cells per number of neutrophils counted. Sensitive but limited by the lack of automation, assay standardization and subjective interpretation.
Cell culture	Blood, Urine, Saliva	Conventional approach where clinical specimens are inoculated onto human fibroblasts, incubated and observed over time. Takes 2–21 days for reporting a result based on a morphological analysis. It is possible to obtain a faster detection of HCMV in culture already 24 h post-infection by using staining with anti-IE antibodies. Highly specific but low sensitivity for HCMV infection.
Polymerase Chain Reaction	Blood, Urine, Saliva, Tissue	Rapid and sensitive method based on amplification of nucleic acids. Targets even low levels of immediate early and late genes or transcripts. Quantitative nucleic acid amplification guides preemptive strategies, monitors response to therapy and is the preferred method for diagnosis of HCMV infection.
Immunohistochemical Staining	Blood, Urine, Saliva, Tissue	Monoclonal or polyclonal antibodies are applied against various HCMV proteins and visualized by dye or fluorescently labelled antibodies or enzyme/polymer labelled secondary antibodies, allowing morphological identification of HCMV in the specimen. It is a highly sensitive and very specific technique. Considered goldstandard for diagnosis in HCMV end-organ diseases.

**Table 2:** Results of HCMV IE and LA positivity in breast cancer specimens and breast cancer metastases, *n* = 111.

	HCMV-IE	HCMV-LA
Grade 0	102 (91.9%)	28 (25.2%)
Grade 1	4 (3.6%)	27 (24.3%)
Grade 2	4 (3.6%)	22 (19.8%)
Grade 3	1 (0.9%)	17 (15.3%)
Grade 4	0	17 (15.3%)
Positive (Total)	9 (8.1%)	83 (74.8%)

## Data Availability

All data is contained within the article, for further information please contact corresponding authors.
